# Wernicke’s encephalopathy — from basic science to clinical practice. Part 1: Understanding the role of thiamine

**DOI:** 10.1177/2045125320978106

**Published:** 2020-12-29

**Authors:** Michael Ott, Ursula Werneke

**Affiliations:** Department of Public Health and Clinical Medicine, Division of Medicine, Umeå University, Umeå, Sweden; Department of Clinical Sciences, Division of Psychiatry, Sunderby Research Unit, Umeå University, Umeå, Sweden

**Keywords:** beriberi, Korsakoff syndrome, magnesium, thiamine, thiamine deficiency, vitamin B1, Wernicke encephalopathy

## Abstract

Wernicke’s encephalopathy (WE) is an acute neuropsychiatric state. Untreated, WE can lead to coma or death, or progress to Korsakoff syndrome (KS) – a dementia characterized by irreversible loss of anterograde memory. Thiamine (vitamin B1) deficiency lies at the heart of this condition. Yet, our understanding of thiamine regarding prophylaxis and treatment of WE remains limited. This may contribute to the current undertreatment of WE in clinical practice. The overall aim of this review is to identify the best strategies for prophylaxis and treatment of WE in regard to (a) dose of thiamine, (b) mode of administration, (c) timing of switch from one mode of administration to another, (d) duration of administration, and (e) use of magnesium along thiamine as an essential cofactor. Evidence from randomized controlled trials and other intervention studies is virtually absent. Therefore, we have to resort to basic science for proof of principle instead. Here, we present the first part of our clinical review, in which we explore the physiology of thiamine and the pathophysiology of thiamine deficiency. We first explore both of these in their historical context. We then review the pharmacodynamics and pharmacokinetics of thiamine, exploring the roles of the six currently known thiamine compounds, their transporters, and target enzymes. We also explore the significance of magnesium as a cofactor in thiamine-facilitated enzymatic reactions and thiamine transport. In the second (forthcoming) part of this review, we will use the findings of the current review to make evidence-based inferences about strategies for prophylaxis and treatment of WE.

## Introduction

Wernicke’s encephalopathy (WE) is an acute neuropsychiatric state. Untreated, WE can lead to coma or death, or progress to Korsakoff syndrome (KS). KS is a dementia characterized by irreversible loss of anterograde memory.^1,2^ Thiamine (vitamin B1) deficiency lies at the heart of this condition. Hence, understanding thiamine is essential for understanding the etiology of WE, its prophylaxis and treatment.

The overall aim of this review is to identify the best strategies for prophylaxis and treatment of WE in regard to (a) dose of thiamine, (b) mode of administration, (c) timing of switch from one mode of administration to another, (d) duration of administration, and (e) use of magnesium along thiamine as an essential cofactor. Evidence from randomized controlled trials and other intervention studies is virtually absent. Therefore, we have to resort to basic science for proof of principle instead. In the first part of our clinical review, we explore the physiology of thiamine and the pathophysiology of thiamine deficiency in their historical context. In the second (forthcoming) part of this review, we will use the findings of the current review to make evidence-based inferences about strategies for prophylaxis and treatment of WE.

## Historical perspective on thiamine

First descriptions of states compatible with thiamine deficiency in form of beriberi appeared in Japan in the 9th century. The concept of WE emerged 1000 years later. Between 1876 and 1878, the neurologist Carl Wernicke at the Charité Hospital in Berlin identified three cases of a hemorrhagic encephalitis, which now bears his name.^3^ The etiology remained unknown at the time; the concept of vitamins emerged only 25 years later. It then took another 30 years to identify the link between thiamine deficiency and WE (Table 1). Whether “thiamin” or “thiamine” is the correct spelling has been debated ever since the name was proposed.^4^ Today, both spellings are used.

**Table 1.** A brief history of thiamine.^5–10^

Year	Milestone
As early as 640	In China and Japan, the term “kakkè” 脚気/かっけ is used to describe what later turns out to be beriberi.^11^
1873–1874	The Dutch naval surgeon Fredrik Johannes van Leent ascribes high mortality from beriberi among Indian crews to their diet, and reduces the incidence of beriberi from 60% to 7% by adding vegetables, meat, bacon, and butter.^12^
1875	The French surgeon and ophthalmologist Charles Jules Alphonse Gayet describes a case of diffuse encephalitis, later thought to have been due to thiamine deficiency.^13^
1876–1878	The German physician Carl Wernicke describes three cases of the hemorrhagic encephalopathy without being able to clarify the etiology.^3^
1884	The Japanese naval medical officer Takaki Kanehiro prevents beriberi effectively by enriching the white rice diet with meat and vegetables, assuming protein deficiency as cause.^6^
1887	The Russian physician Sergei Korsakoff describes cases of a dementia with anterograde amnesia without being able to clarify the etiology.^14^
1886	The Dutch government sends professor Cornelis Pekelharing and neurologist Cornelis Winkler to Jakarta (at that time Batavia, part of the Dutch East Indies) to investigate beriberi. They tentatively conclude that beriberi is of bacterial origin.^15^ They return home and leave their assistant Christiaan Eijkman to isolate the causative organism.
1890–1897	Eijkman conducts experiments in chicken and demonstrates that a diet of polished rice, not bacteria, is associated with a beriberi-like illness.^16^ He confirms the causative association of polished rice and beriberi in an observational study with Adolphe Vorderman in 279,623 prison inmates.^17^ He postulates the presence of an antidote for the disease in the silver skin of rice.
1901	Gerrit Grijns, successor to Eijkman in the Indonesian laboratory, concludes that there is rather a protective substance in rice, but even meat and vegetables, essential to maintain the function of the nervous system. “Partial starvation”, for example, absence of that micronutrient, which is destroyed by cooking, leads to beriberi.^18^
1906	Eijkman establishes that the protective substance is water-soluble.^19^
1910–1912	The Polish scientist Casimir Funk identifies an “antineuritic substance”, which he called beriberi “vitamine” (vita: life; amine: nitrogen containing compound).^20^
1920	The British biochemist Jack Drummond suggests the term vitamin (without “-e” and the distinction of vitamins by name of the alphabet.^21^
1926	The Dutch chemist Barend Jansen together with his colleague Willem Donath isolates pure vitamin B1.^22^
1929	Eijkman, jointly with Sir Frederick Hopkins, receives the Nobel Prize for Physiology or Medicine for the discovery of vitamins.^23^
1936	The American chemist Robert Williams synthesises thiamine and later licenses the production process to Merck.^24^
1937	Several European countries accept the name “aneurine” for vitamin B1. Williams suggests the name “thiamin”, derived from the Greek “theion” = sulfur and amine.^25^
1941	A. C. P. Campbell and Ritchie Russell at the Scottish Mental Hospital’s Laboratory in Edinburgh suggest vitamin B1 deficiency as a cause of Wernicke’s encephalopathy.^10^
1963	The Bread and Flour Regulations 1963 require the addition of vitamin B1 to non-wholegrain bread in Britain.^26^
1974	In collaboration with the local baker, general practitioner Max Kamien fortified the bread with thiamine, niacin and iron in a small town in the Australian outback, eliminating vitamin B deficiency signs in Aboriginal people.^27^ Thiamine fortification of flour becomes mandatory in Australia 1991.^28^
1999	Thiamine transporter 1 (SLC19A2) is cloned by several groups.^29–32^ Organic cation transporter (OCT) 3 is shown to have thiamine as substrate.^30^
2000	Thiamine transporter 2 (SLC19A3) is identified and characterized.^33^
2001	The reduced folate carrier (SLC19A1) is shown to transport phosphorylated thiamine compounds.^34^
2011	Prion proteins are shown to bind thiamine.^35^
2014	The human colonic thiamine pyrophosphate transporter (SLC44A4) is identified.^36^ OCT 1 is recognised as thiamine transporter in the liver.^37^ The role of multidrug and toxin extrusion proteins (MATE) and OCT 2 in renal excretion of thiamine is discovered.^38^ A putative thiamine transporter (SLC35F3) is characterized.^39^

## Manifestations of thiamine deficiency

Thiamine deficiency can arise from (a) reduced intake, (b) impaired absorption, (c) inability to convert thiamine to its biologically active form, or (d) excessive elimination. Alcohol use disorder accounts for about 50% cases of WE. Bariatric surgery, consuming illnesses, malabsorption syndromes, and hyperemesis are other examples of possible causes.^40,41^ We will discuss such causes in more detail in the second, forthcoming, part of this review.

Thiamine deficiency is often divided in two different disease entities, Wernicke-Korsakoff-syndrome and beriberi. Beriberi has been categorized further according to organ involvement into dry, wet, and gastrointestinal beriberi. As WE and beriberi overlap, it may be more accurate to use thiamine deficiency as an umbrella term, to then be specified further to reflect the respective clinical problem (Table 2).^42^ In this way, six different thiamine deficiency states can be described, WE, KS, dry and wet beriberi, Shoshin beriberi, and thiamine-deficiency-associated lactic acidosis. Gastrointestinal beriberi is probably a manifestation of thiamine-deficiency-mediated lactic acidosis. Thiamine deficiency occurs not only in adults; both beriberi and WE have been described in children.^43,44^

**Table 2.** Thiamine deficiency states.^42,45–50^

	WE	Korsakoff’s syndrome	Dry beriberi	Wet beriberi	Shoshin beriberi	Thiamine deficiency mediated lactic acidosis/gastrointestinal beriberi
Organ system predominantly affected	Central nervous system		Peripheral nervous system	Cardiovascular system		Ubiquitous
Onset	Acute	Chronic	Chronic	Subacute	Acute	Acute
Symptoms classically described	Nystagmus, ophthalmoplegia, ataxia, confusion	Memory loss, anterograde and retrograde, amnesia, apathy	Symmetrical peripheral neuropathy with both sensory and motor impairments, mostly of the distal extremities	Hyperdynamic heart failure, oedema, ↓ peripheral vascular resistance, hypotonia, metabolic acidosis		Anorexia, nausea, vomiting and abdominal pain, Kussmaul breathing ↑ lactate
Etiology	↓ thiamine intake ↓ thiamine absorption ↑ thiamine elimination	As WE or as or sequel of WE without current thiamine deficiency	↓ thiamine intake	↓ thiamine intake + ↓ folate?	↓ thiamine intake + ↓ folate?	↓ thiamine intake ↓ thiamine absorption ↑ thiamine elimination

WE, Wernicke’s encephalopathy.

## Dietary thiamine

The recommended daily thiamine intake depends on age, sex, and calorie and carbohydrate intake. As a rule of thumb, thiamine intake should be at least 0.4 mg/1000 kcal. The recommended dietary thiamine intake is 1.4 mg for adult males and 1.0 mg for adult females. In pregnancy, daily thiamine requirements rise to 1.6–1.8 mg daily. In the United Kingdom (UK), the average daily intake from food sources is about 1.5 mg.^51^ If the daily thiamine intake falls below 0.2 mg/1000 kcal, urinary excretion becomes low. Clinical symptoms of thiamine deficiency may then emerge within 8 weeks.^52^ Thiamine can be found in many foodstuffs including meat, wholegrain products, fortified grain products, pulses, and some fruits. Yeast extracts contain most thiamine, and sugar is devoid of thiamine. As a general rule, unless fortified, processed foods contain less thiamine than comparable non-processed food stuffs (Table 3). Of all meats, pork has the highest thiamine content. Food preparation involving heat can lead to a 20% thiamine loss.

**Table 3.** Comparable foods of higher and lower thiamine content.^53,54^

Item	Thiamine (mg/100 g)	Item	Thiamine (mg/100 g)
Higher thiamine content^a^		Lower thiamine content^a^	
Ham	0.80	Turkey slices	0.05
Pork chop, grilled, lean	0.78	Chicken breast, coated, baked	0.10
Bacon, streaky, fried	0.75	Pork sausages, grilled	traces
Cornflakes fortified	0.60	Cornflakes unfortified	traces
Peas, frozen	0.26	Peas, canned in water, reheated, drained	0.09
Bread, whole meal, average	0.25	Cakes from “healthy eating” ranges	0.06
Bread, white, average	0.24	Sponge cake, home made	0.08
Chapati, made without fat	0.23	Rice cakes	0.02
Peanuts, dry, roasted	0.18	Potatoes crisps, fried in sunflower oil	0.09
Potatoes, old, boiled	0.18	Potato chips, from fast food outlet	0.07
Spaghetti, dried, whole wheat, cooked	0.11	Spaghetti, dried, white, cooked	0.08
Rice, brown, wholegrain, cooked	0.11	Rice, long grain, boiled	traces
Lentils, red, boiled	0.11	Chickpeas, canned in water, reheated, drained	0.05
Oranges	0.11	Apples	0.04
For comparison			
Yeast extract	4.10	Sugar	0.00

a Estimates based on several food samples in each category.

## Thiamine compounds

**Figure 1.**
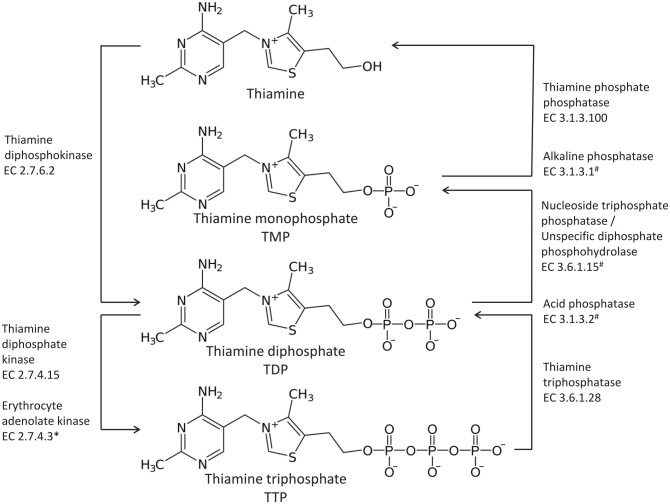
Thiamine compounds. *In red blood cells. ^#^Thiamine phosphate esters are hydrolyzed by various phosphatases and nucleotide diphosphatases.

There are six known thiamine compounds, free thiamine, thiamine monophosphate (TMP), thiamine diphosphate (TDP), adenosine thiamine diphosphate (ATDP), thiamine triphosphate (TTP), and adenosine thiamine triphosphate (ATTP)^5,55^ (Figure 1).

TDP is also referred to as thiamine pyrophosphate (TPP). In humans, free thiamine, and TMP account for 5–15% of the total thiamine. TDP is the principal biologically active form, and accounts for 80–90% of total thiamine. TDP presents in high concentrations in skeletal muscle, liver, heart, kidneys, and brain. The remaining three components, TTP, ATTP, and ATDP, account for only 1% of total thiamine in humans.^55^ Whole blood thiamine levels can vary significantly between populations (Table 4).

**Table 4.** Whole blood thiamine concentrations in four populations.^56–60^

*n*	Sex	Age range (year)	Free (nmol/l)	TMP (nmol/l)	TDP (nmol/l)	TTP (nmol/l)	Total (nmol/l)	Ratio phosphorylated thiamine (TMP + TDP + TMP)/free thiamine
Japan^a^								
509	M	21–80	7 (2–18)	17 (5–47)	124 (70–229)	0 (0–4)	150 (89–262)	22 (9–58)
460	F	18–70	6 (2–17)	16 (4–60)	114 (63–200)	0 (0–3)	139 (80–235)	22 (8–58)
Norway 1991^b^								
15	M	32–54	33.4 (10.4)	10.9 (5.1)	165.0 (40.4)	<2		
15	F	23–60	29.6 (10.0)	9.7 (2.3)	121 (29.6)	<2		
The Netherlands^b^								
65			4.3 (1.9)	4.1 (1.6)	120 (17.5)	<4		
					Belgium^b^			
7			4 (3)	10 (4)	138 (33)	13 (4)		
					The Netherlands^c^			
98							115 (70–185)	

a Mean (95% confidence interval).

b Mean (standard deviation).

c Mean (range)

F, female; M male, TDP, thiamine diphosphate; TMP, thiamine monophosphate; TTP, thiamine triphosphate.

Approximately 75% of whole blood thiamine is stored in the erythrocytes, 15% in leukocytes, and 10% in plasma.^61^ The compounds can be phosphorylated or dephosphorylated as required. The enzymes necessary are under genetic control (Table 5).

**Table 5.** Enzymes required for the conversion of thiamine compounds.^1,55,62–66^

HGNC approved gene symbol	Cytogenic location	Transporter/enzyme expressed	Reaction	Magnesium requirement	Pathologies associated with genetic change
TPK1 protein network	7q35	Thiamine diphosphokinase/Thiamine diphosphotransferase EC 2.7.6.2	Free thiamine → TDP	Divalent cations, best activation with Mg^2+^	Thiamine metabolism dysfunction syndrome 5: onset of acute encephalopathic episodes in early childhood^67^
		Thiamine-diphosphate kinase EC 2.7.4.15	TDP → TTP	Mg^2+^ required by several animal organisms, but not shown for humans	
AK1	9q34.11	Erythrocyte adenolate kinase EC 2.7.4.3	TDP → TTP in red blood cells	Mg^2+^ required	Defect associated with hemolytic anaemia^68^
THTPA protein network	14q11.2	Thiamine- triphosphatase/TTP Hydrolase EC 3.6.1.28	TTP → TDP	Mg^2+^ required	
ALPI	2q27.1 1p36.12	Alkaline phosphatase EC 3.1.3.1	TDP → TMP TMP → free thiamine	Intestine. Mg^2+^ used as a cofactor	
Multiple	Multiple	Acid phosphatase EC 3.1.3.2	TTP → TDP TDP → TMP	Mg^2+^ used as a cofactor	
Not available	Not available	Nucleoside-triphosphate phosphatase/unspecific diphosphate phosphohydrolase EC 3.6.1.15	TDP → TMP	Mg^2+^ not required	
Not available	Not available	Thiamine phosphate (mono) phosphatase EC 3.1.3.100	TMP → free thiamine	Mg ^2+^ enhanced membrane-bound activity 1.7-fold, soluble enzyme independent of Mg ^2+^ (based on animal experiments)	

ALPI, alkaline phosphatase, intestinal; EC, Enzyme Commission; HGNC, HUGO Gene Nomenclature Committee; Mg^2+^, bivalent magnesium cation; TDP, thiamine diphosphate; THTPA, thiamine triphosphatase; TMP, thiamine monophosphate; TPK1, thiamine pyrophosphokinase; TTP, thiamine triphosphate.

Free thiamine and TMP are the thiamine transport compounds that deliver thiamine to and from the cells. TDP and TTP are the compounds that unfold the biological action. The different forms are constantly converted into each other to maintain thiamine availability.

## Thiamine metabolism

Mechanism of thiamine uptake and transport through the body has crucial implications for the understanding of the etiology of WE and treatment rationale. Humans cannot synthesize thiamine but depend on two exogenous sources: dietary and bacterial thiamine.^69^ Transport of thiamine and conversion of various thiamine compounds into each other are under genetic control. These mechanisms require further clarification (Table 4).

## Thiamine actions and implications for deficiency states

Thiamine plays a central role in energy metabolism. Thiamine is also implicated in the physiology of neurotransmission.

### Energy metabolism

**Figure 2.**
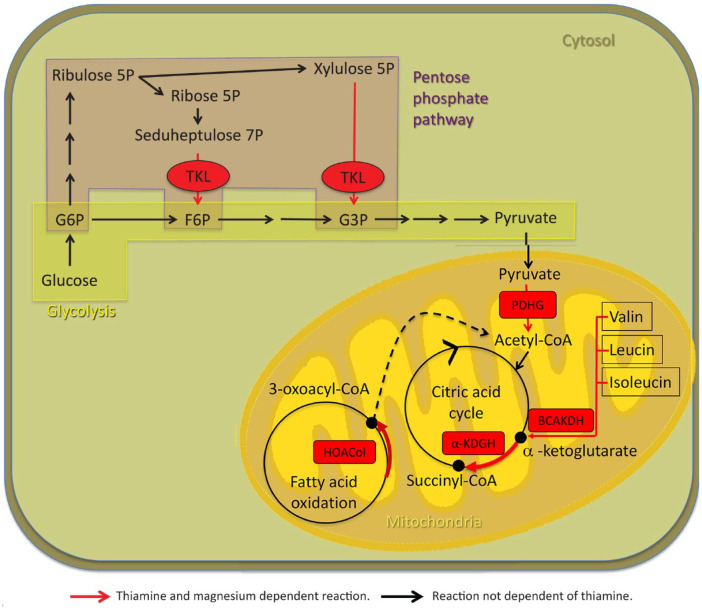
Thiamine- and magnesium-dependent metabolic pathways. Red arrows, thiamine- and magnesium-dependent reactions; black arrows, reactions not dependent on thiamine. α-KGDH, α-ketoglutarate dehydrogenase; BCAKDH, branched chain α-keto acid dehydrogenase; F6P, d-fructose 6-phosphate; G3P, d-glyceraldehyde 3-phosphate; G6P, d-glucose 6- phosphate; HOACol, 2-hydroxyacyl CoA lyase 1; PDHG, pyruvate dehydrogenase complex; TKL, transketolase.

TDP acts as an important cofactor in glucose, fatty acid, and protein metabolism, as well as adenosine triphosphate (ATP) generation. Thus, TDP is a critical cofactor for transketolase (TK), pyruvate dehydrogenase (PDHG), α-ketoglutarate dehydrogenase (α-KGDH), branched chain α-keto acid dehydrogenase E1 (BCAKDH E1), and 2-hydroxyacyl CoA lyase 1 (HOACoL).^5,70–76^ These enzymes hold key roles in mitochondrial energy (ATP) generation in body and nerve cells, nucleic acid synthesis, carbohydrate, fatty acid metabolism, and amino acid metabolism. If these enzymes do not function properly, energy metabolism becomes impaired and oxidative stress increases. At the same time, undesirable compounds may accumulate if chemical reactions are re-routed. For instance, if α-KGDH is impaired, glutamate is produced instead of succinyl CoA. If PDHG is impaired, lactate is produced instead of acetyl CoA^70^ (Table 6, Figure 2).

**Table 6.** Key metabolic enzymes that use thiamine pyrophosphate as a cofactor.^5,62,65,70–75,77^

Enzyme	HGNC approved gene symbol	Cytogenic location	Subcellular location	Pathway/function	Magnesium as a further cofactor	Functions of metabolic products and resulting other pathways intermediates	Clinical implication in deficiency states
TK EC 2.2.1.1	TKT	3p21.1	Cytosol, extracellular exosome, nucleus, peroxisome, vesicles	Pentose phosphate pathway	Yes	Substrates in the glycolytic pathways Generation of d-ribose-5-P → Nucleotide synthesis → RNA/DNA synthesis NADPH as a reducing agent for fatty acid and acetylcholine synthesis, maintenance of myelin sheaths Aromatic amino acid synthesis	↓ energy supply ↓ nucleotide synthesis ↓ fatty acid synthesis → facilitating demyelination ↑oxidative stress Amino acid imbalance
TK protein 1 EC 2.2.1.1	TKTL 1	Xq28	Cytosol, nucleus	As TK	Yes	May structurally alter TPP to change TPP affinity for TK^78^	Unclear
PDHG EC 1.2.4.1	PDHA1	Xp22.12	Mitochondrion, nucleus, PDHG complex (GO:0045254)^a^	Glycolysis (rate-limiting co-factor)	Yes	Fatty acid, ketone bodies and acetylcholine synthesis, maintenance of myelin sheaths Generation of Acetyl CoA Generation of citrate, the first component in the TCA cycle	↓ fatty acid synthesis → demyelination ↓ Energy (ATP) production Leigh phenotype
α-ketoglutarate dehydrogenase α-KGDH/OGDC EC 1.2.4.2	OGDH	7p13	Mitochondrion, nucleus, OGDC (GO: 0045252)^a^	TCA (citric acid) cycle	Yes	Energy production (ATP) Generation of succinyl CoA	↑ nitric oxide and peroxidase activity →oxidative stress ↓ energy production Lactate acidosis and ↑focal extracellular glutamate → oedema → excitotoxicity → BBB permeability → neuronal death
BCKDH E1 subunit α EC 1.2.4.4	BCKDHA	19q13.2	Mitochondrion	Degradation of branched chain amino acids, valine, leucine and isoleucine, facilitating the oxidative decarboxylation step	Yes	Isobuturyl CoA, α-methylbuturyl CoA, isovaleryl CoA → acetyl CoA, acetoacetate, succinyl CoA → Fatty acid, ketone bodies and acetylcholine synthesis, maintenance of myelin sheaths	↓ fatty acid synthesis → demyelination ↑ Valine, leucine and isoleucine and corresponding α-ketoacids → maple syrup urine disease
2-hydroxyacyl CoA lyase 1 (HACL1) EC 4.1.-.-	HACL1	3p25.1	Cytosol, peroxisomes	Oxidation of 3-methyl branched fatty acids such as phytanic acid and 2-hydroxy fatty acids (α-oxidation)	Yes	Formate → CO_2_	Peroxisome biogenesis defects → impeding the breakdown of certain nutrients including amino acids degeneration and β-oxidation of fatty acids ↑ phytanic acid → Refsum disease

a Note that this term represents a location and not a function.

ATP, adenosine triphosphate; BCKDH, branched chain α-keto acid dehydrogenase; Co A, coenzyme A; EC, Enzyme Commission numbers; GO, gene ontology; HGNC, HUGO Gene Nomenclature Committee; NADPH, nicotinamide adenine dinucleotide phosphate (hydrogenated, i.e. reduced form); ODGC, oxoglutarate dehydrogenase complex; P, phosphate; PDHG, pyruvate dehydrogenase; TCA cycle, tricarboxylic acid cycle; TK, transketolase; TPP, thiamine pyrophosphate.

Thiamine deficiency therefore disrupts energy metabolism and ATP production. α-KGDH and TK are two key enzymes in the pathophysiology. Decreased α-KGDH activity can develop within 4 days of thiamine deficiency.^1^ Decreased α-KGDH activity results in increased oxidative stress, lactate acidosis, excitotoxicity, for instance through glutamate accumulation, inflammation and disturbed blood brain barrier (BBB) permeability, cerebral edema, and, ultimately, neuronal death.^71,79^ Decreased TK can develop within 1 week of thiamine deficiency.^1^

### Signal transmission

Thiamine phosphates have even non-enzymatic actions on neurotransmitters and hormones through second messengers. Whereas other B-vitamins activate the adenyl cyclase system, thiamine activates the guanyl cyclase system. cGMP is an important second messenger for peptide hormones and nitric oxide (NO), facilitating smooth muscle relaxation, mediating penile erection, regulating vascular and airway tone, peristalsis, and insulin secretion.^5,75^ TDP also acts as a cofactor for PDGH and facilitates acetylcholine synthesis. Thiamine even modulates choline neurotransmission non-enzymatically. This can be deducted from the observation that the metabolic thiamine antagonist oxythiamine can increase acetylcholine release.^74,80^ The role of TDP in the modulation of glutamate neurotransmission arises from its impact on α-KGDH. Thiamine may also regulate the activity on the astrocyte glutamate aspartate transporters.^81^ These transporters are under genetic control. Defects may result in insufficient clearance of glutamate and hence to an increase of interstitial glutamate. This could lead to hyperexcitability, neurotoxicity and cell death as possible consequences.^81–83^

Finally, thiamine may also modulate other neurotransmitters such as serotonin.^1^ Ultimately, much of what we know is based on in vitro and animal experiments. Our understanding of thiamine function in the brain remains limited.^55,74^

## The journey of thiamine through the body

### Thiamine transport in and out of cells: general principles

For the human organism, thiamine is an extremely valuable substance due to its central role in many metabolic processes (Table 6). As thiamine cannot be synthesised by humans, it is crucial to secure a steady supply to the cell. This involves maximizing uptake and minimizing loss. In most cells, different systems of transporters are available to facilitate uptake into the cell and into the mitochondria (Table 7). The kidneys are fine-tuned to either reabsorb or excrete thiamine depending on the actual thiamine plasma concentration. A further mechanism of facilitating thiamine uptake into the cells may involve prion proteins (PrP).^84^ PrP are membrane-anchored proteins, particularly abundant in neuronal cells in vertebrates. It has been shown that thiamine binds to PrP.^35^ The thiamine transporters are quickly saturated when extracellular thiamine concentrations exceed the physiological range. Thiamine transporter functions partly overlap. For instance, the reduced folate carrier (RFC/SLC19A1) can take over transport from Thiamine transporter 1 (ThTR-1). General cation-transporters, which are not specific to thiamine, can also transport thiamine at higher concentrations. Such include transporters belonging to the organic cation-transporter (OCT) and multidrug and toxin extrusion proteins (MATE) family (Table 7). This partial overlap of thiamine transporters may be the reason why not all thiamine transporter deficiencies are associated with pathologies.

**Table 7.** Thiamine transporters.^1,55,63–65,67,85^

HGNC approved gene symbol	Cytogenic location	Transporter/enzyme expressed	Pathologies associated with genetic change	Comment/possible defect
SLC19A1	21q22.3	Folate transporter (FOLT)/reduced folate carrier (RFC)		Can act as transporter for TDP (Zhao 2001) and/or TMP in some tissues when ThTR-1 fails.^86^
SLC19A2	1q24.2	Thiamine transporter 1 (ThTR-1)	Thiamine metabolism dysfunction syndrome 1 (Thiamine-responsive megaloblastic anaemia syndrome/Rogers syndrome)	High-affinity transporter for thiamine intake.^30^ Association of the megaloblastic anaemia and with diabetes mellitus and hearing loss.^87^ Three genetic variants found in 25 patients with alcohol-associate WE, only one of these variants found in healthy controls.^88^
SLC19A3	2q36.3	Thiamine transporter 2 (ThTR-2)	Thiamine metabolism dysfunction syndrome 2 (biotin- or thiamine-responsive encephalopathy type), Leigh syndrome, Infantile lethal encephalopathy	High-affinity transporter for thiamine intake. SLC19A3 associated disorders present as neurological disorders that bear some features similar to WE.^89,90^ Association with seizures and brain atrophy in early infancy.^67^
SLC22A1	6q25.3	Organic cation transporter 1 (OCT 1)		Primary hepatic uptake transporter of thiamine.^91^ Intestinal uptake of thiamine?^92,93^ Expressed on luminal side of renal tubular cells.^94^
SLC22A2	6q25.3	Organic cation transporter 2 (OCT 2)		Predominantly expressed in kidney, but even neurons^95,96^ and microvessels of the blood-brain barrier.^97^ Renal tubular secretion/reabsorption of thiamine.^38^ Expressed on basolateral side of renal tubular cells.^94^
SLC22A3	6q25.3	Organic cation transporter 3 (OCT 3)		Intestinal uptake of thiamine.^92,93^ OCT3 in human liver cells showed a higher affinity but lower capacity compared with OCT1^98^ but a lower affinity than ThTR. Expressed in microvessels of the blood-brain barrier^97^ and various brain regions.^99,100^ Expressed on basolateral side of renal tubular cells.^101^
SLC25A19	17q25.1	Mitochondrial TDP carrier.	Leigh syndrome, Thiamine metabolism dysfunction syndrome 3 (microcephaly Amish type), Thiamine metabolism dysfunction syndrome 4 (bilateral striatal degeneration and progressive polyneuropathy type).^102^	Mediates thiamine uptake into mitochondria. Believed to be important for brain development and interfere with α-KGDH activity.
SLC35F3	1q42.2	Putative thiamine transporter		Association with arterial hypertension.^39^
SLC44A4	6p21.33	Choline transporter like protein	Autosomal dominant deafness	Acts also as a TDP transporter (human TDP transporter) in the colon and facilitate uptake of microbiota-generated thiamine. Thiamine created by colon microbiota exists in form to TDP.^103,104^ Autosomal dominant deafness has been attributed to defects in the choline transporter function.
SLC47A1	17p11.2	MATE1		Expressed on luminal side of renal tubular cells.^105^ Renal tubular secretion of thiamine.^38^ Expressed in microvessels of the blood-brain barrier.^97^
SLC47A2	17p11.2	MATE2-K		Specifically expressed in the kidney^106^ but even in microvessels of the blood-brain barrier.^97^ Renal tubular secretion of thiamine.^38^

HGNC, HUGO Gene Nomenclature Committee; MATE1, multidrug and toxin extrusion protein; MATE2-K, kidney-specific multidrug and toxin extrusion protein; SLC, solute carrier family [number] family [letter, number] TKT; TDP, thiamine diphosphate; TMP, thiamine monophosphate; TTP, thiamine triphosphate.

### From food to enterocyte

**Figure 3.**
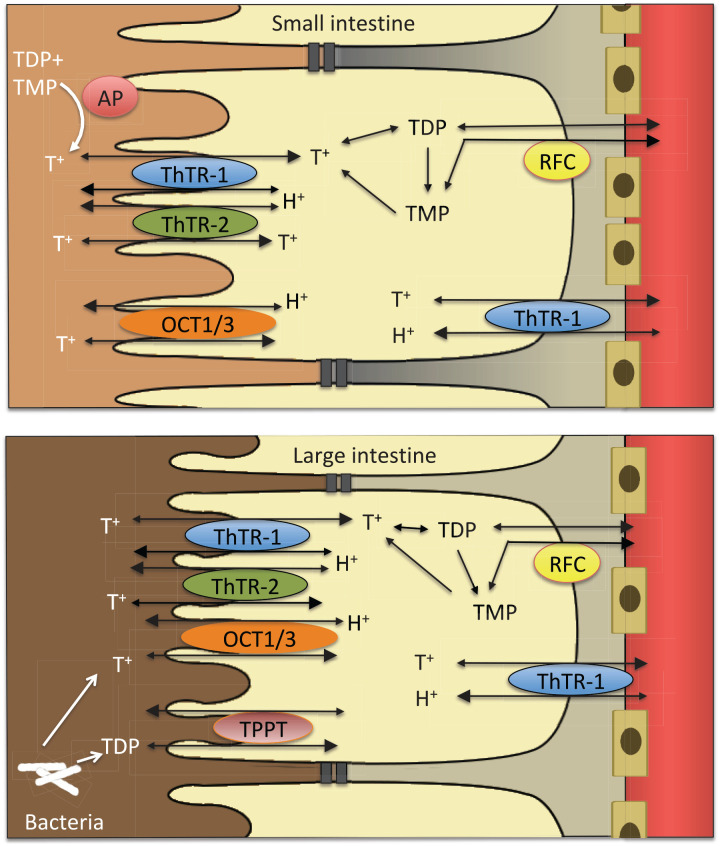
From food to blood. OCT, organic cation transporter; RFC, reduced folate carrier; T^+^, free thiamine; TDP, thiamine diphosphate; ThTR, thiamine transporter; TMP, thiamine monophosphate; TTP, thiamine triphosphate.

Dietary thiamine exists mainly in phosphorylated forms. Exogenously acquired thiamine is thought to be first de-phosphorylated to free thiamine by gastrointestinal phosphatases.^107^ Free thiamine is then absorbed in the small intestine, preferentially in the jejunum. There are two transport mechanisms, active and passive (Figure 3). The active transport system involves ThTR-1 and ThTR-2.^108^ The passive transport is proportional to the thiamine concentration in the intestinal lumen.^109^ Where epithelial and endothelial linings are made up by tight junctions, only small molecules can pass through by simple passive diffusion. As thiamine is a relatively large molecule, its passive transport most likely occurs in the form of *facilitated* diffusion, along an electrochemical gradient via protein channels.^110^ This suggests that, within limits, passive thiamine transport is a non-saturable process.^109,111^ Active and passive thiamine transport may operate simultaneously.^112^ The active transport follows a Michaelis–Menten kinetic and seems to be saturated at a concentration of 2-2.5 µmol/l.^113–115^ As shown in lines of heterogenous human epithelial colorectal adenocarcinoma cells (Caco-2 cells), active transport has evolved to maximize thiamine uptake in scenarios of low availability.^116^ Animal experiments have shown that thiamine uptake in the small intestine can be increased dramatically during thiamine deficiency. However, during chronic alcohol use, thiamine uptake is reduced. In the presence of alcohol, expression of both ThTR-1 and ThTR-2 diminished significantly.^117^

An alternative source of thiamine is bacterial. Bacteria flora in the large intestine may synthesize both free thiamine and TDP.^36,103,104^ Previously, it has been assumed that this colonic thiamine cannot be used. Now, based on animal cell culture experiments, it has been suggested that microbiota generated TDP is taken up into the colonocytes by a human TDP transporter. At present it remains unclear how much colonic thiamine contributes to physiological functions of TDP across the body (Figure 3).^36,69,104^

### From enterocyte to blood

**Figure 4.**
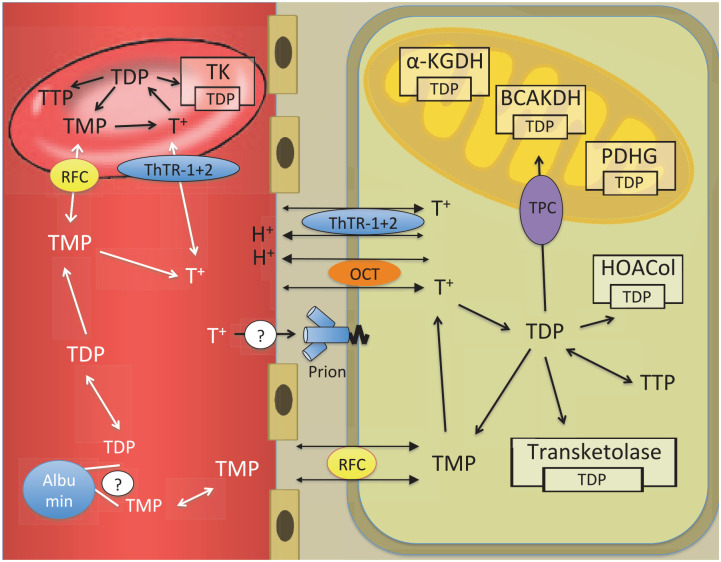
From blood to cell. α-KGDH, α-ketoglutarate dehydrogenase; BCAKDH, branched chain α-keto acid dehydrogenase; HOACol, 2-hydroxyacyl CoA lyase 1; OCT, organic cation transporter; PDHG, pyruvate dehydrogenase; RFC, reduced folate carrier; T^+^, free thiamine; TDP, thiamine diphosphate; ThTR, thiamine transporter; TK, thiamine kinase; TMP, thiamine monophosphate; TPC, mitochondrial thiamine pyrophosphate carrier; TTP, thiamine triphosphate.

In the enterocyte, as in any other cell, free thiamine can be phosphorylated directly to TDP. The enterocyte uses a part of this TDP for its own metabolic needs. The rest is broken down to TMP and free thiamine. Free thiamine and TMP are then transported out of the cell into the plasma.^64,112^ The release of thiamine into plasma has been ascribed to ThTR-1,^108^ but other transporters may be involved. As an organic cation, thiamine can be transported by organic cation/antiport systems into the enterocyte.^107^ From there, organic cation transporter proteins (OCT) 1 and 3 can transport thiamine into the blood.^92,112^ OCT 1 and 3 can take up thiamine in concentration ranges from 10 to 500 µmol/l (Figure 4).^112^ This may be the mechanism behind the passive thiamine transport at high concentrations. However, it remains currently unclear how far this extracellular “flooding” of cells with free thiamine leads to higher intracellular levels of pharmacologically active phosphorylated thiamine forms. Alcohol can impair the *active* intestinal transport mechanism.^69,117^ However, alcohol does not seem to affect *passive* uptake of thiamine at high doses.^114,118^

### From blood to tissue

Once in the plasma, free thiamine and TMP are distributed throughout the body. Phosphorylated thiamine is bound partly to plasma proteins.^119^ Both free thiamine and TMP can enter the cell. Free thiamine in the cationic form crosses the cell membrane with help of ThTR-1.^108^ The mechanism by which TMP crosses the cell membrane is not well described. RFC (SLC19A1) has been implicated (Table 4).^86,120^ Once free thiamine enters the cell, it is phosphorylated to TDP directly without generation of TMP as an intermediary. The responsible enzyme is thiamine diphosphokinase, which catalyzes the following reaction: free thiamine + ATP → TDP + AMP. A small part can be phosphorylated further to TTP. TTP can be dephosphorylated to TDP, TDP to TMP, and TMP to free thiamine (Figure 4).^55,64^

In a study of thiamine kinetics in six healthy volunteers,^121^ large increases in thiamine blood concentration after intravenous (i.v.) injection reflected increases in extracellular plasma concentrations. Only a little free thiamine was stored in the blood cells. Further, only a small amount was phosphorylated to TDP. At the same time, thiamine was eliminated seven times faster from the plasma than blood cells. These findings hint at thiamine phosphorylation rather than thiamine transport being the rate-limiting factor for TDP availability.

### From blood to brain

Data from small post-mortem studies indicate brain thiamine concentrations between 25.1 and 32.5–36.2 pmol/mg protein.^58,122^ These are lower concentrations than reported in rodents or other primates. TDP is by far the most prominent compound. The above concentrations translate to 283 nmol thiamine/100 g fresh brain tissue.^55^ Standard textbooks report “thiamine” concentrations in the brain between 0.14 mg and 0.44 mg/100 g fresh brain tissue.^123,124^ However, these data are not referenced. For an average brain of 1300 g, this would amount to 1.82–5.72 g or 5.6–17.5 µmol. About 2% of all available thiamine is transported into the brain.^117^ As any other solute, thiamine cannot freely enter the brain. Instead, all solutes need to cross either the BBB or the blood-cerebrospinal fluid barrier (BCSFB).^120^ The BBB is the barrier between cerebral blood vessels and brain tissues. The BCSFB is the barrier between the blood and the CSF on the one hand and the CSF and brain tissues on the other. The choroid plexus forms the interface between the blood/CSF component. The arachnoid membrane forms the interface between the CSF/brain tissue component.^110,125^ Within the brain, there is no barrier between the extracellular space and the CSF.^110,126^ Substance transport over the BCSFB is not thought to contribute relevantly to the metabolic needs of the brain.^127^

The anatomical structure of the BBB with tight cellular junctions limits the possibility of thiamine passing into the brain by simple passive diffusion. Indeed, has it been suggested that, “normally,” less than 10% of B vitamins is transferred into the brain by simple passive diffusion.^128^ How exactly thiamine enters the brain remains unclear. The BBB consists of vascular endothelium, pericytes, and astrocyte end-feet. The space between endothelial cells is rendered impermeable by tight junctions. Therefore, contrary to permeable endothelial cells in other parts of the body, substances cannot by-pass the endothelial cells in the brain. Instead, they have to pass through the endothelial cells. Like neurons and glial cells, BBB-forming cells have specific solute carriers.^129^ Free thiamine seems to enter the brain via active transport, most likely involving ThTR-2. The transport system is half-saturated at normal plasma concentration of 0.1–0.3 µmol/l. Lack of the other major thiamine transporter, ThTR-1, leads to thiamine-responsive megaloblastic anaemia syndrome but not to WE. Therefore, ThTR-1 may not be essential for thiamine uptake into the brain.^120^ Parallel to the active carrier-mediated process, a non-saturable process also exists at higher concentrations.^111,130,131^ Thiamine may also be taken up passively via facilitated diffusion.^120,132^

TMP may possibly enter the brain via active transport involving the reduced folate carrier.^120^ This transport system is half-saturated at normal plasma concentration of 25 µmol/l. Overall, there are about 10 µmol thiamine in the brain. The rate of thiamine turnover in the brain is about 60–100% per day. This suggests that thiamine homeostasis in the brain is managed tightly to render a steady state between thiamine entering and leaving the brain.^120^ Thus, administration of large amounts of thiamine for medicinal purposes may not necessarily lead to increased thiamine concentrations in the brain.^56,110^ An animal experiment reported in 1968 supports this assumption. Rats fed a thiamine-deficient diet developed overt encephalopathy, in which thiamine brain concentration fell to less that 20% of normal. Increasing thiamine to only 26% of normal concentration reversed symptoms to an essentially normal neurologic state.^133^ In humans, reversibility of symptoms is variable. Prompt treatment can reverse symptoms as long permanent damage and cell death has not occurred.^2,71^

### Body stores

The body can store about 30 mg of thiamine.^70,74^ Again, most of this can be expected to be intracellular in the form of TDP. The concentrations are highest in heart, skin, kidneys, adipose tissue, lung, and colon.^134^ It remains unclear how long these stores last in circumstances of thiamine deficiency. One study investigated the urinary excretion of thiamine in eight young men. These consumed a 2800 kcal diet that provided 400 g of carbohydrates and 0.11–0.18 mg thiamine a day. This corresponded to 10% of the recommended nutritional intake (RNI) of thiamine. Urine thiamine decreased to <50 µg a day within 6 days and became undetectable on the 18th day of thiamine depletion.^135^ Another study of three volunteers found a half-time excretion for thiamine of 9.5, 13, and 18.5 days.^136^ These studies suggest that thiamine depletion may occur within approximately 2-3 weeks of thiamine deficiency.

## Deactivation and metabolism

Thiamine can be broken down by two thiaminases: type I and II. These cleave thiamine into its pyrimidine and thiazole moieties.^137^ In humans, thiaminase activity is negligible under normal conditions. However, thiamine deficiency can occur when thiaminase-containing foods are ingested excessively or not processed properly. Thiaminase I is found in fish, shellfish, ferns, and some bacteria. Thiaminase II is found in some bacteria.^138^ Thiaminases are usually heat-labile; they can be destroyed through cooking.^70^ Thiaminase I in nardoo (*Marsilea drummondii*), an Australian fern can, however, withstand high temperatures. One historical account of beriberi due to thiaminase poisoning stems from the Burks and Wills expedition to cross Australia from coast to coast from 1860 to 1861. Of the four participating European men, only one survived. During their journey, the men began eating nardoo-based flour, preparing it the European way instead of the Aborigine way. It is speculated that they did not soak the fern sufficiently long in water to diminish the activity of nardoo thiaminase 1.^139^

Polyhydroxyphenols, caffeic acid, phenols, flavonoids, and tannins, can also serve as anti-thiamine factors. Such polyhydroxyphenols are found, for instance, in coffee, tea, betel nuts, blueberries, blackcurrants, Brussels sprouts, and red cabbage. They destroy thiamine by an oxidative process transforming thiamine to non-absorbable thiamine disulfide.^140^ Polyhydroxyphenols are heat-stable components and cannot be destroyed through cooking.^70^ Therefore, excessive ingestion can lead to thiamine deficiency.^141^

## Recycling and elimination in the kidney

**Figure 5.**
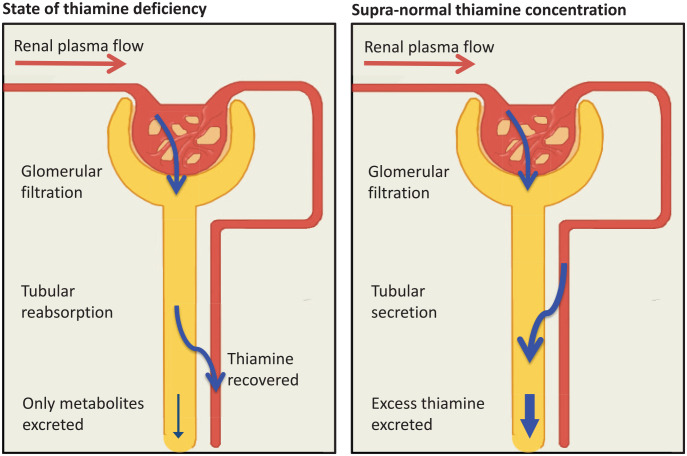
From blood to urine or urine to blood.

In humans, a multitude of metabolites have been identified by radioactive labelling of the pyrimidine or thiazole moiety of thiamine.^136^ Studies in rats demonstrated up to 22 different thiamine metabolites identifiable in the urine.^142–144^ The kidneys can largely adapt their handling of free thiamine to the current plasma concentration (Figure 5). Therefore, free thiamine is eliminated or reabsorbed renally, depending on thiamine status. However, thiamine metabolites cannot be reabsorbed in the kidney. Paradoxically, the amount of metabolites excreted is not decreased in a state of thiamine deficiency.^135^

In the glomerulus, thiamine is filtrated freely, like any other small solute. Glomerularly filtrated thiamine is then processed in the proximal tubule. Thiamine stored in blood cells or bound to protein cannot be filtrated. Data on plasma protein binding is again conflicting. Thom *et al.* found that, under physiological conditions, up to 30% of plasma thiamine may be bound to albumin (10% TMP, 20% TDP).^119^ These thiamine compounds may bind to albumin via the phosphate moiety of the molecule. At thiamine concentrations over 119.5 µmol/l, albumin binding decreases to 2%. Weber *et al.*, however, suggested that plasma thiamine was not bound to protein.^145^

Under physiological conditions, up to a concentration of 200 nmol/l, thiamine is reabsorbed to minimize excretion. Phosphorylated thiamine, mostly in the form of TMP, is dephosphorylated to free thiamine in the tubule.^145^ ThTR-1, ThTR-2, and OCT1, expressed in the brush border membrane mediate thiamine uptake from urine into the tubular cells. ThTR-1 as well as OCT2 and 3 in the basolateral membrane mediate thiamine uptake from tubular cells into the blood. ThTR transporters have a much higher affinity than OCT. They are also saturated at lower concentrations. Principally, all transporters are bidirectional.^38,85,146–148^

In thiamine deficient states, thiamine transporters are upregulated.^149^ Ziporin *et al.* showed that urinary thiamine excretion could decrease to undetectable levels.^135^

**Figure 6.**
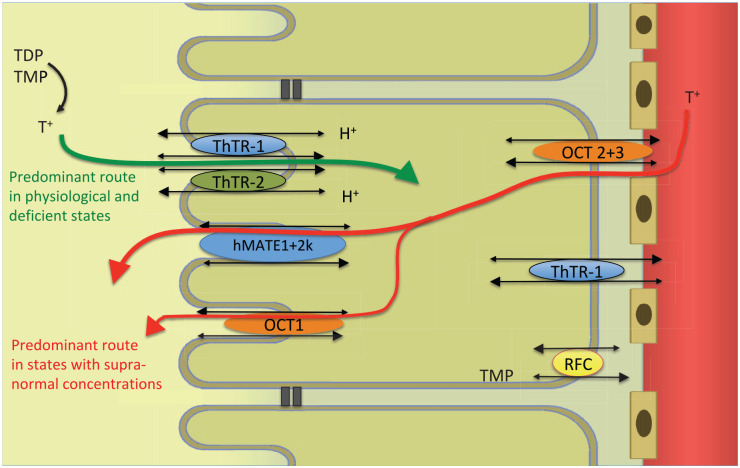
Renal regulation of thiamine. hMATE, human multidrug and toxin extrusion protein; OCT, organic cation transporter; RFC, reduced folate carrier; T^+^, free thiamine; TDP, thiamine diphosphate; ThTR, thiamine transporter; TMP, thiamine monophosphate.

In states of thiamine excess, for example, achieved through pharmacological dosing, thiamine is eliminated completely by the kidneys.^145^ Elimination is increased by switching from reabsorption to active secretion. In that case, thiamine that has not been filtrated in the glomerulus is excreted via the renal tubular cells. Thiamine directly inhibits ThTR-1 mediated uptake.^30^ In such circumstances, the ThTR mediated pathway may be reversed from thiamine uptake to secretion.^147^ Additionally, the kidneys can eliminate thiamine through two types of cation transporters: OCTs and multidrug and toxin extrusion proteins (MATEs). Thiamine enters the renal tubular cells from the blood through OCT 1 and 2 located in the basolateral membrane.^148^ From there, thiamine is excreted across the brush border membrane into the urine through MATE1 and MATE2-k.^38,150–152^ This mechanism can lead to complete plasma thiamine elimination from all the blood passing through the kidneys (renal blood flow). The elimination amounts to five times the glomerular filtration rate (Figure 6).^145^

## Magnesium as cofactor for thiamine

Magnesium is an alkaline earth metal with an atomic weight of 24.31. In the body, magnesium occurs mostly ionized as the bivalent cation Mg^2+^. Mg^2+^ is the fourth most abundant cation in the body after sodium, potassium, and calcium, and the second most abundant intracellular cation after potassium.^153–155^ Mg^2+^ is a cofactor to more than 600 enzymatic reactions.^156^ Mg^2+^ is implicated in the energy metabolism of macronutrients, oxidative phosphorylation, protein and nucleic acid synthesis, neuro-muscular signal conduction, and regulation of cell membrane permeability.^154,155^ Despite its crucial contribution to physiology, magnesium is relatively under-researched compared with other nutrients, such iron or calcium. Therefore, clinicians may be much less vigilant to possible states of magnesium deficiency.^156^ Part of the problem is that magnesium deficiency is difficult to assess by a simple blood test; more than 99% magnesium is intracellular.^154,157^

## Significance of magnesium for thiamine function

A significance of magnesium for alcohol-associated WE was first suggested in 1964.^158^ Along with thiamine, magnesium has since turned out to be an essential cofactor to several key metabolic enzymes (Table 5) that govern glucose, fatty acid, and protein metabolism as well as ATP generation. Magnesium is also required as cofactor for thiamine transport and conversion of the various thiamine compounds into each other (Table 6). Without magnesium, thiamine cannot function properly. This implies that magnesium deficiency can impair thiamine activity. But we do not know how substantial magnesium deficiency has to become to trigger clinical symptoms of thiamine deficiency (*cf.* magnesium deficiency below).

### Dietary magnesium

Recommendations for daily intake depend on age and sex. They vary somewhat internationally. In the UK, the recommended daily magnesium intake is 300 mg for adult males and 270 mg for adult females.^51^ In the United States (US), the recommended daily intake is slightly higher, 420 mg for men and 320 mg for women aged between 31 and 50 years. No extra magnesium is required during pregnancy. However, an additional 50 mg per day is required for women who breastfeed to compensate for magnesium losses in breast milk.^53^ Magnesium is found in nearly all foods. As a central component of chlorophyll, magnesium is particularly common in leafy vegetables. Whole grain cereals, nuts, and yeast extracts are also rich sources of magnesium.^51,53,157^

## Magnesium metabolism

Mg^2+^ absorption and excretion depend on nutritional uptake, intestinal absorption, and renal capacity for reabsorption. Magnesium can be taken up anywhere across the intestine. It has been suggested that the duodenum takes up 11% of all the magnesium absorbed, the jejunum 22%, the ilium 56%, and the colon 11%.^157^ Of all absorbed Mg^2+^, between 20% and 70% are excreted again in the feces.^156^ The remainder is distributed throughout the body. About 50–60% are stored in the bone, 20–30% in muscles and 20–25% in other organs. An average adult stores about 24 g Mg^2+^ in the body. Only 0.8% of magnesium is found in the blood, 0.3% in serum, and 0.5% in erythrocytes. There is a constant exchange between Mg^2+^ stored and Mg^2+^ in the blood. The kidney can filtrate about 10% of bodily Mg^2+^, corresponding to a renal filtration rate of 2.4 g/day. Between 5% and 70% can, however, be reabsorbed and re-entered into the redistribution cycle.^153,156,157^

### Magnesium deficiency

Magnesium deficiency may manifest itself in neurological symptoms, such as neuromuscular hyperexcitability and weakness. Magnesium deficiency is also associated with electrocardiographic (ECG) changes and arrhythmias, hypoparathyroidism, and vitamin D deficiency. There are also biochemical changes, including hypocalcaemia, hypokalaemia, and metabolic alkalosis.^159,160^ However, unless severe, symptoms of magnesium deficiency can be difficult to spot. Normal magnesium serum concentration lies in a range from 0.7 to 1.0 mmol/l. Magnesium deficiency may occur when the serum magnesium concentration falls below 0.66 mmol/l. Yet, clinical symptoms may become observable only at levels below 0.5 mmol/l.^161^ As magnesium is over 99% intracellular, a normal magnesium serum concentration does not exclude deficiency. Such normo-magnesemic magnesium deficiency may occur in patients with chronic harmful use of alcohol.^162^

Magnesium deficiency can occur in three contexts: (a) decreased uptake or absorption, (b) increased gastrointestinal or renal elimination, or (c) shift from the extracellular to the intracellular space.^163,164^ Reduced magnesium uptake can occur in the context of total nutritional deficiency. It can also occur with one-sided nutrition with low magnesium content. Inflammatory bowel diseases and treatment with proton pump inhibitors such as omeprazole are associated with reduced magnesium absorption. Increased gastrointestinal magnesium elimination occurs for instance in the context of excessive vomiting or laxative abuse. Renal magnesium wasting can also occur in the context of treatment with loop or thiazide diuretics, cisplatin, amphotericin, aminoglycosides, foscarnet, cyclosporine, and tacrolimus.^155,164^ Increased renal elimination occurs when tubular reabsorption becomes impaired, for instance in the context of osmotic diuresis or some renal diseases. Such include interstitial nephritis, Gitelman, Bartter, and Fanconi syndromes. Shifts from extracellular to intracellular space occur in settings of refeeding, treatment of diabetic ketoacidosis or other metabolic acidosis, hungry bone syndrome or pancreatitis. Alcohol dependency is associated with several risk factors for magnesium deficiency, including poor nutrition, gastrointestinal problems associated with proton pump inhibitor use and reduced absorption, increased diuresis, excessive urinary excretion, and vomiting.^160,165^

## Therapeutic implications

Thiamine is a key factor in human energy metabolism and an important contributor to neurotransmitter functions. These fundamental roles explain why thiamine deficiency can lead to devastating consequences such as WE. Thiamine stores in humans are limited, and thiamine homeostasis depends on external thiamine sources. For many thiamine-mediated reactions, even magnesium is required as a cofactor. In spite of the enormous significance of thiamine and its dependency on magnesium, surprisingly few studies have examined strategies for prophylaxis and treatment of deficiency states. In the absence of clinical evidence, a thorough understanding of the basic science behind thiamine can assist in formulating treatment strategies, with a rationale resting on pharmacodynamic and pharmacokinetic concepts. In this first part of this review, we have explored the basic science behind thiamine. In the forthcoming second part of this review, we will examine current guidelines for prophylaxis and treatment of WE in light of our understanding of the basic science behind thiamine.
